# Influenza Infection Complicated by Ventricular Fibrillation, Cardiac Arrest, and Torsades De Pointes: A Case Report

**DOI:** 10.7759/cureus.106327

**Published:** 2026-04-02

**Authors:** Chathushki Bandara, Amit Bhambri

**Affiliations:** 1 Emergency Medicine, Endeavor Health Swedish Hospital, Chicago, USA

**Keywords:** cardiac arrest, cardiac arrhythmia, influenza, torsades de pointes, ventricular fibrillation

## Abstract

Patients seeking evaluation in the emergency department (ED) for symptoms of an upper respiratory tract infection is a common occurrence. Emergency medicine physicians have adapted to the role of providing advice and therapies for lower-acuity presentations when outpatient services are limited. In most cases, a thorough history and physical examination help guide appropriate testing and subsequent treatment for a possible viral or bacterial etiology. However, it is uncommon for upper respiratory illnesses to present as life-threatening complications.

We present a case of a 25-year-old healthy male who initially developed common upper respiratory symptoms, including coughing, subjective fevers, and fatigue. Two days after developing his symptoms, his partner found him unconscious in bed. After Emergency Medical Services (EMS) arrived at the scene, they determined that the patient was in ventricular fibrillation (Vfib) cardiac arrest. The patient’s ED course was complicated by additional episodes of Vfib and pulseless Torsades de Pointes (TdP). Remarkably, testing revealed that he was positive for influenza A. This unique case serves as a reminder that seemingly benign upper respiratory tract infections can still lead to deadly complications, even in young, healthy individuals.

## Introduction

Every year, influenza places a significant burden on our health. Of the 40 million cases of influenza reported in the United States during the 2023-2024 season, there were 470,000 people who required hospitalization and 28,000 deaths [1]. Although most influenza cases only involve a course of upper respiratory symptoms, some patients may develop more life-threatening complications from the virus.

Typically, influenza is perceived as a respiratory virus limited to a short course of coughing, fevers, and malaise. There is growing evidence to suggest that the influenza virus itself could increase the risk of developing acute cardiovascular events. Such events include myocardial infarction, cardiomyopathy, heart failure exacerbation, myocarditis, and pericarditis [2]. The link between influenza infection and cardiovascular morbidity is not clearly understood. There are several proposed mechanisms, including direct cardiomyocyte infection and systemic inflammatory responses secondary to infection [3]. Cardiac arrhythmias are another consequence of myocardial insult caused by the influenza virus. Influenza infection may contribute to subsequent cardiac muscle inflammation and electrolyte disturbances, propagating malignant cardiac arrhythmias. This case presents an otherwise healthy adult male who was infected with influenza and presented to the emergency department (ED) with ventricular fibrillation (Vfib) and Torsades de Pointes (TdP), both of which are deadly alterations to the heart's inherent electrical conduction. 

## Case presentation

A 25-year-old male with no past medical history was brought to the ED by Emergency Medical Services (EMS) for cardiac arrest. The patient developed “flu-like” symptoms, including generalized fatigue, subjective fevers, and coughing, over the previous two days after exposure to his partner, who was also suffering from similar symptoms. Prior to ED arrival, the patient was found unconscious in bed, seemingly after an episode of emesis. The partner immediately called 911 and was instructed by EMS personnel to start cardiopulmonary resuscitation (CPR) in the field. Minutes later, when EMS arrived at the scene, the patient was pulseless, and the cardiac monitor depicted Vfib. The patient was immediately defibrillated at 200 Joules, with return of spontaneous circulation (ROSC) in the field.

**Figure 1:**
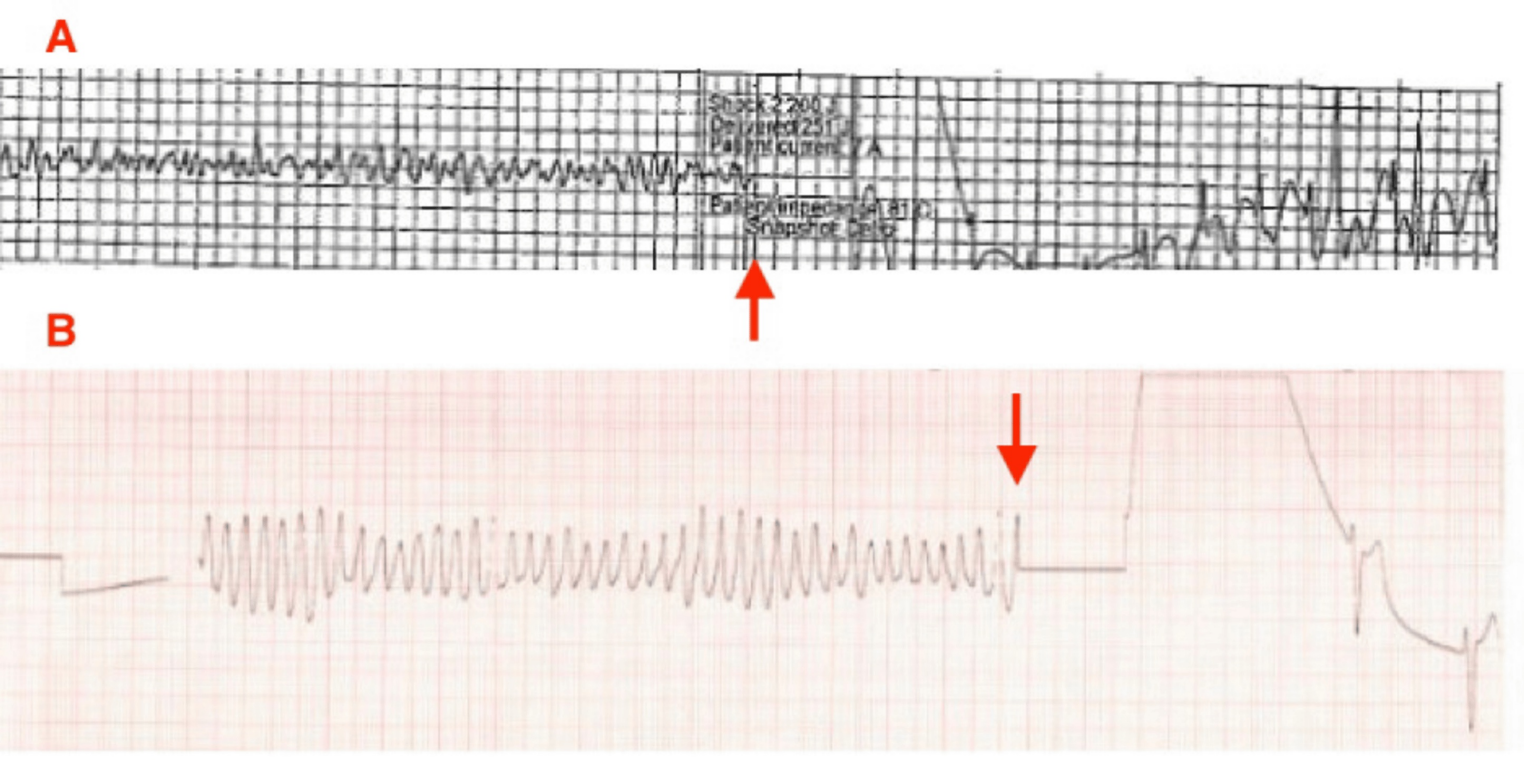
A) Patient's telemetry strip showing ventricular fibrillation preceding red arrow, defibrillation marked by red arrow, followed by cardiac electrical restabilization. B) Telemetry strip from episode of pulseless Torsades de Pointes with oscillatory twisting of QRS complex amplitude from isoelectric line, followed by defibrillation at red arrow.

After achieving ROSC, the patient was then brought to the ED at time 15:30. His initial ED vitals were blood pressure 126/82 mmHg, heart rate of 118 beats per minute (sinus tachycardia), respiratory rate of 31 breaths per minute, and temperature of 99.6°F. He did not open his eyes to voice or painful stimuli, verbalized incomprehensible sounds, and withdrew from pain. He was intubated for airway protection, with oxygen saturation at 100% on the ventilator set at FiO₂ 100%. His labs were drawn around time 15:46. Minutes after he was intubated, the patient developed Vfib again, which fortunately responded to immediate defibrillation (Figure 1A). However, while awaiting laboratory results over the next 30 minutes, the patient developed frequent, persistent episodes of pulseless TdP, visualized on the telemetry monitor, despite administration of intravenous magnesium at time 16:00 and intravenous amiodarone (300 mg) at time 16:03. With each persistent episode of pulseless TdP, he was subsequently defibrillated at 200 Joules, after which a palpable pulse was regained immediately (Figure 1B). These episodes of pulseless TdP occurred innumerable times, despite multiple defibrillations, amiodarone, and magnesium. After discussion with the on-call cardiologist, the patient was started on an isoproterenol drip at around time 16:27, which mitigated any further cardiac dysrhythmias. He was ultimately stabilized on isoproterenol infusion, sedated on fentanyl and propofol drips while intubated, and admitted to the Intensive Care Unit (ICU) for continued evaluation and management.

**Figure 2:**
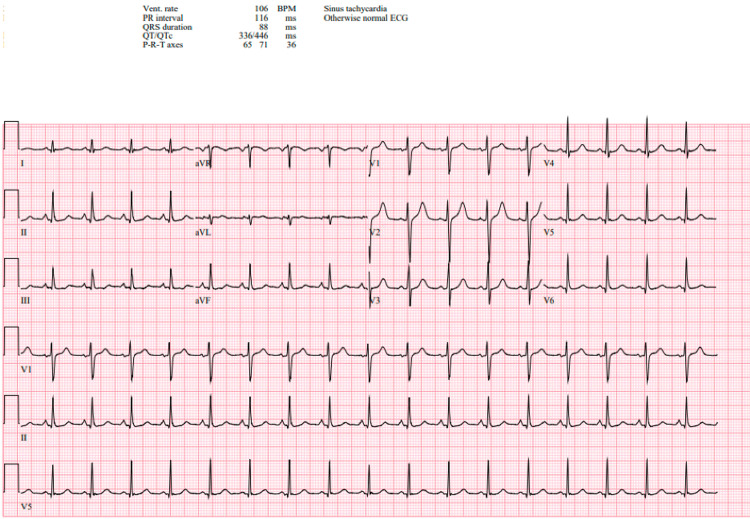
Electrocardiogram

The patient's laboratory values are listed in Table 1. These blood samples were drawn during the frequent episodes of pulseless TdP and prior to the patient's stabilization. The labs were remarkable for leukocytosis of 17.22 × 10³/µL, with an elevated procalcitonin of 1.04 ng/mL. Blood chemistry showed normal values for sodium (139 mEq/L), potassium (3.4 mEq/L), and calcium (9.7 mg/dL). Potassium was repleted with 20 mEq of intravenous potassium chloride in the ED. An arterial blood gas (ABG) suggested metabolic acidosis, with pH 7.24, PCO₂ 37 mmHg, and HCO₃ 15.4 mEq/L. Magnesium was elevated at 2.9 mg/dL. Initial troponin was 262.27 ng/L, and after four additional sets, troponin later peaked at 755 ng/L. Brain natriuretic peptide (BNP) was normal. Lactic acid was 4.6 mmol/L. Coagulation studies were normal. Urine drug screen, alcohol, acetaminophen, and salicylate levels were negative. He was Influenza A positive. Computed tomography (CT) chest (pulmonary embolism (PE) protocol) showed small areas of ground-glass opacities in the lower lobes that could reflect pneumonia. Once the patient was stabilized on isoproterenol infusion, his electrocardiogram (EKG) in the ED showed sinus tachycardia with normal axis, normal PR interval, normal QRS interval, and a slightly prolonged QTc of 446 milliseconds (Figure 2).

**Table 1:** Initial laboratory values drawn in the Emergency Department WBC: white blood cells; RBC: red blood cells; Hct: hematocrit; MCV: mean corpuscular volume; MCH: mean corpuscular hemoglobin; MCHC: mean corpuscular hemoglobin concentration; CO₂: bicarbonate; BUN: blood urea nitrogen; AST: aspartate aminotransferase; ALT: alanine aminotransferase; PCO₂: partial pressure of carbon dioxide; PO₂: partial pressure of oxygen; HCO₃: bicarbonate, arterial; BNP: brain natriuretic peptide; PT: prothrombin time; INR: international normalized ratio; PTT: partial thromboplastin time; TSH: thyroid-stimulating hormone

Laboratory Test	Patient Value	Reference Range
WBC	17.22	4.5-11.0 × 10^3^/uL
RBC	5.09	4.20-6.00 × 10^6^/uL
Hemoglobin	16.3	13.5-18.0 g/dL
Hct	48.4	40.0-54.0%
MCV	95.1	80.0-95.0 CU Microns
MCH	32.1	27.0-31.0 pg
MCHC	33.7	32.0-36.0 g/dL
Platelet Count	178	130-500 × 10^3^/uL
Neutrophil %	82.6	40.0-74.0%
Lymphocyte %	11.9	19.0-48.0%
Monocyte %	3.8	3.4-9.0%
Eosinophil %	0.5	0.0-7.0%
Basophil %	0.2	0.0-1.5%
Glucose	210	70-99 mg/dL
Sodium	139	135-147 mEq/L
Potassium	3.4	3.4-5.3 mEq/L
Chloride	105	96-108 mEq/L
CO_2_	24	22-32 mEq/L
Anion Gap	10	0-16 mEq/L
BUN	13	7-23 mg/dL
Creatinine	1.0	0.4-1.3 mg/dL
Calcium	9.7	8.6-10.5 mg/dL
Magnesium	2.9	1.6-2.6 mg/dL
Albumin	4.4	3.5-5.0 g/dL
Total Bilirubin	0.6	0.2-1.4 mg/dL
AST	158	5-34 U/L
ALT	194	5-50 U/L
Alkaline Phosphatase	43	46-116 U/L
pH Arterial	7.24	7.35-7.45
PCO_2_ Arterial	37	35-45 mmHg
PO_2_ Arterial	472	80-100 mmHg
HCO_3_ Arterial	15.4	22-26 mEq/L
CO_2_ Total Arterial	16.5	mEq/L
Base Excess Arterial	-11.2	mEq/L
O_2_ Saturation	98.5	94.0-97.0%
Troponin I	262.27	0.0-53.5 ng/L
Troponin I 2 HR	552.51	0.0-53.5 ng/L
Troponin I 4 HR	688.18	0.0-53.5 ng/L
BNP	19.9	0.0-100.0 pg/mL
Lactic Acid	4.6	0.5-2.2 mmol/L
Procalcitonin	1.04	0.03-0.49 ng/mL
PT	14.5	0.8-1.2 s
INR	1.1	0.8-1.2
PTT	32.5	24.0-37.2 s
Alcohol	< 10.0	< 10.0 mg/dL
Salicylates	< 3.0	2.0-29.0 mg/dL
Acetaminophen	< 2.0	0.0-199.0 ug/mL
Urine Drug Screen	Negative	Negative
TSH	0.430	0.4-4.00 µIU/mL
COVID-19	Negative	Negative
Influenza A	Positive	Negative
Influenza B	Negative	Negative

While hospitalized, the patient underwent further inpatient testing, including an echocardiogram that showed a left ventricular ejection fraction of 50%-55% with normal global left ventricular systolic function and normal segmental wall motion. His CT cardiac angiography showed no evidence of obstructive disease and a total plaque volume of 0 mm³ by HeartFlow Plaque analysis. Cardiac magnetic resonance imaging (MRI) did not reveal any findings to suggest myocardial fibrosis or scarring. Two days after admission to the ICU, the patient was gradually weaned off all sedation drips and extubated. On day 2 of admission, the patient was transitioned from the isoproterenol drip to the amiodarone drip as directed by the cardiac electrophysiologist. On day 4 of admission, he was transitioned to oral amiodarone. As recommended by the cardiac electrophysiologist, the patient was fitted for a wearable external defibrillator and started on amiodarone 200 mg orally twice daily for four weeks, followed by 200 mg orally daily thereafter for a total of three months. The patient's electrolytes, including sodium, potassium, and magnesium, trended normally for the total six days of admission. His troponin peaked on day 2 of admission at 755.56 ng/L. A repeat EKG prior to discharge home showed that the QTc remained at 447 ms. He was scheduled for outpatient follow-up to discuss plans for implantable cardioverter defibrillator (ICD) placement for secondary prevention of future similar events.

## Discussion

Although most cases of influenza resolve after a short course of upper respiratory symptoms, this case presents an uncommon complication. A healthy young adult, with no family history of heart rhythm disease or premature cardiac death, developed Vfib cardiac arrest and TdP while infected with influenza.

Remarkably, the only abnormal tests for this patient, suggesting an organic cause of his presentation, were the positive influenza A swab and the development of secondary pneumonia on chest CT imaging. The elevated white blood cell (WBC) and procalcitonin reflect this infection. The magnesium elevation was likely secondary to the supplementary intravenous magnesium sulfate given while the patient was in TdP. His troponin elevations were likely multifactorial, including the combination of CPR and multiple defibrillations.

Notably, this patient’s cardiac dysrhythmia was responsive to isoproterenol. Isoproterenol is a non-selective beta-1 and beta-2 adrenergic receptor agonist. While its approved indication is for heart block, it can be used off-label for refractory TdP secondary to acquired long-QT syndrome [4]. Isoproterenol decreases the QT interval, therefore further stabilizing the cardiac membrane [5]. This patient’s QT/QTc was only mildly prolonged at 336/446 milliseconds (normal QTc in a healthy male is <440 milliseconds); however, the value was obtained from his EKG performed after he was stabilized on isoproterenol.

The patient’s inpatient testing, including echocardiogram, CT cardiac angiography, and cardiac MRI, did not reveal preexisting cardiac disease. Knowing this, it is difficult to understand why this specific patient developed a cardiac complication from influenza infection. Structural heart disease may not be apparent in some individuals who have underlying genetic mutations [6]. For example, in arrhythmogenic right ventricular hypertrophy (ARVH), cardiac tissue is gradually replaced with fatty scar tissue. These patients are initially asymptomatic, with little to no cardiac structural changes, but, with disease progression, can propagate unexplained arrhythmias [7].

What may have predisposed this patient to develop an acute cardiovascular event from influenza A remains uncertain. The patient's medical record documents receiving several vaccinations in the past, including COVID-19; however, his influenza vaccination status is unknown. Although it cannot be concluded whether his presentation resulted from lacking protective immunological barriers to the influenza virus, the case sheds some light on the importance of following appropriate vaccination guidelines. According to the CDC, only 44.2% of children less than age 17, and 45.5% of adults aged 18 and above, received the influenza vaccine for the current 2025-2026 flu season [8]. The vaccine may not completely shield an individual from becoming infected by the virus due to the nature of high mutation rates. However, it is the simplest and most effective way to prevent potentially life-threatening complications, even in healthy individuals [9].

## Conclusions

This case was an atypical presentation of a common illness. Although influenza infection usually resolves after a course of upper respiratory symptoms, physicians should still be aware of its dangers, particularly with acute cardiovascular events. A previously healthy 25-year-old male suffered from Vfib, cardiac arrest, and TdP while infected with influenza A. Fortunately, this patient was resuscitated in the ED through a combination of several therapies, including defibrillation, magnesium, amiodarone, and ultimately, isoproterenol. He was eventually discharged from the hospital without any neurologic sequelae. At discharge, he was prescribed amiodarone and a wearable external cardiac defibrillator, with outpatient plans to discuss ICD placement. There are limitations to the case, including QTc values obtained only after the patient was stabilized on antiarrhythmic medications, unknown medication or electrolyte disturbances contributing to arrhythmia, and an unknown genetic channelopathy workup. Despite having an unremarkable medical and family history, patients can still succumb to malignant arrhythmias during influenza infection.
